# Lambl's Excrescences Associated With Left Frontal Ischemic Stroke: A Case Report

**DOI:** 10.7759/cureus.9371

**Published:** 2020-07-24

**Authors:** Sherif Elkattawy, Muhammad Atif Masood Noori, Anuraag Sah, Syed M Hasan Kazmi, Dhaval Desai

**Affiliations:** 1 Internal Medicine, Rutgers New Jersey Medical School/Trinitas Regional Medical Center, Elizabeth, USA; 2 Internal Medicine, Dow Medical College, Karachi, PAK; 3 Internal Medicine, Rutgers New Jersey Medical Center/Trinitas Regional Medical Center, Elizabeth, USA; 4 Medicine, Dow University of Health Sciences (DUHS), Karachi, PAK; 5 Cardiovascular Disease, Jersey Shore University Medical Center, Neptune, USA; 6 Internal Medicine, Trinitas Regional Medical Center, Elizabeth, USA

**Keywords:** cardioembolic, lambls excrescence, stroke

## Abstract

Lambl's excrescences (LEs) are thin filiform strands of connective tissue found on the closure line of valves. Their exact etiology is unknown, and most of them are typically asymptomatic. We present a case of a 33-year-old African American male with a history of right upper extremity weakness that resolved on its own within a few minutes. On physical examination, no focal neurologic deficit was observed. MRI brain showed a tiny curvilinear focus of restricted diffusion with subtle T2-FLAIR hyperintensity in the left frontal/insular region, indicating a recent cortical infarct. Transesophageal echocardiogram (TEE) was performed as a part of cryptogenic stroke workup, which showed LE on aortic valve leaflet tips. Our patient had elevated blood pressure (BP) on arrival; however, in LE's presence, the embolic phenomena of stroke cannot be excluded. In conclusion, this case adds to a limited number of cases with such cardioembolic phenomena, which will help in further illustrating and highlighting the need for more prospective studies to establish a causal relationship between LE and cardioembolic strokes.

## Introduction

Ischemic strokes account for 87% of all strokes in the United States as per the CDC, whereas cardioembolic strokes account for nearly 20% of all ischemic strokes [1-2]. In 1856, a Bohemian physician, Vilém Dušan Lambl, first described the presence of filiform lesions in aortic valve leaflets that were named after him [3]. He further characterized them as tiny mobile, filiform fronds that arise on the line of valve closure and result from endothelial damage due to valvular wear and tear [3-8]. Lambl’s excrescences (LEs) are mostly asymptomatic; however, few cases are described with a causal association with the cardioembolic stroke [9]. We present a 33-year-old African American male with no past medical history who presented with right arm weakness, found to have an acute left frontal stroke, and LE on transesophageal echocardiogram (TEE).

## Case presentation

A 33-year-old African American male with no past medical history presented to the ED with complaints of right arm weakness. As per the patient he was going to work and as he stepped out of his car he attempted to grab his wallet with his right hand but was unable to. He reported he was unable to move his hand for one to two minutes with complete resolution of symptoms after that. The patient denied vision changes, dizziness, lightheadedness, headaches, nausea/vomiting, difficulty or change in hearing, changes in his speech or difficulty swallowing, extremity weakness/paralysis, or any change in sensation. Additionally, he denied any loss of consciousness, change in mental status, visual/auditory auras, or urinary/bowel incontinence. He reported that at work on the day of admission, he was in the middle of a conversation with a coworker and felt "not himself" as he felt "less calm" than usual. As a result, his blood pressure (BP) was checked at that time, which was 179/122 mmHg with a heart rate (HR) of 93/minute. As per the patient, his last annual examination was about one week ago in which his BP was within normal limits.

**Figure 1:**
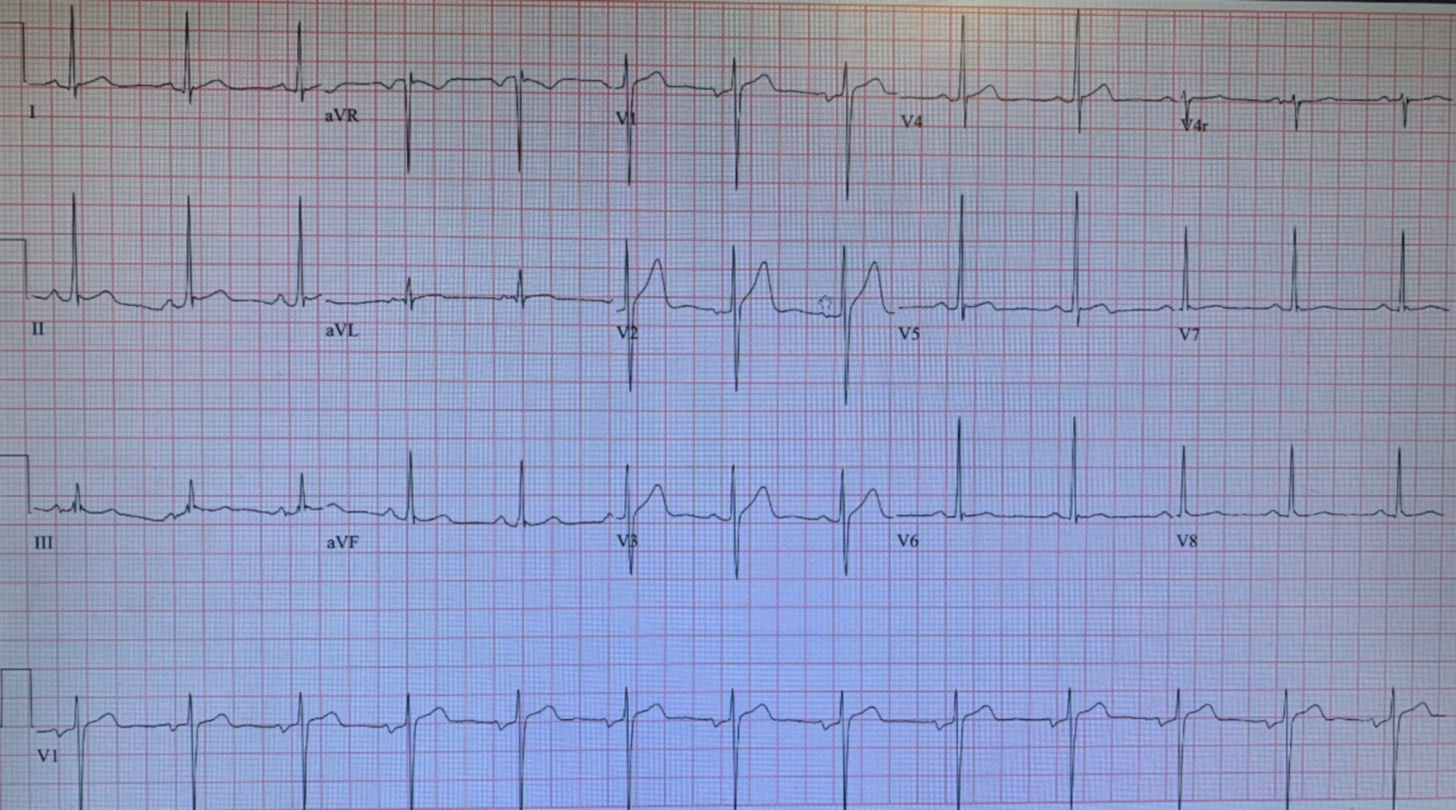
ECG showing high amplitude R waves in V5-V6, indicating left ventricular hypertrophy. ECG, electrocardiogram

In the ED, he was afebrile with elevated BP 174/120 mmHg for which he received labetalol 10 mg IV push that reduced his BP down to 144/96 mmHg. Physical examination was nonfocal, and initial electrocardiogram (ECG) was positive for high amplitude R waves in V5-V6, indicating left ventricular hypertrophy as seen in Figure 1.

CT scan of head without contrast ruled out any acute intracranial hemorrhage; CT angiography of head and neck with IV contrast showed no large vessel occlusion. The patient was loaded with aspirin 325 mg, started on high-intensity statin, and admitted to medical floors for further management.

**Figure 2:**
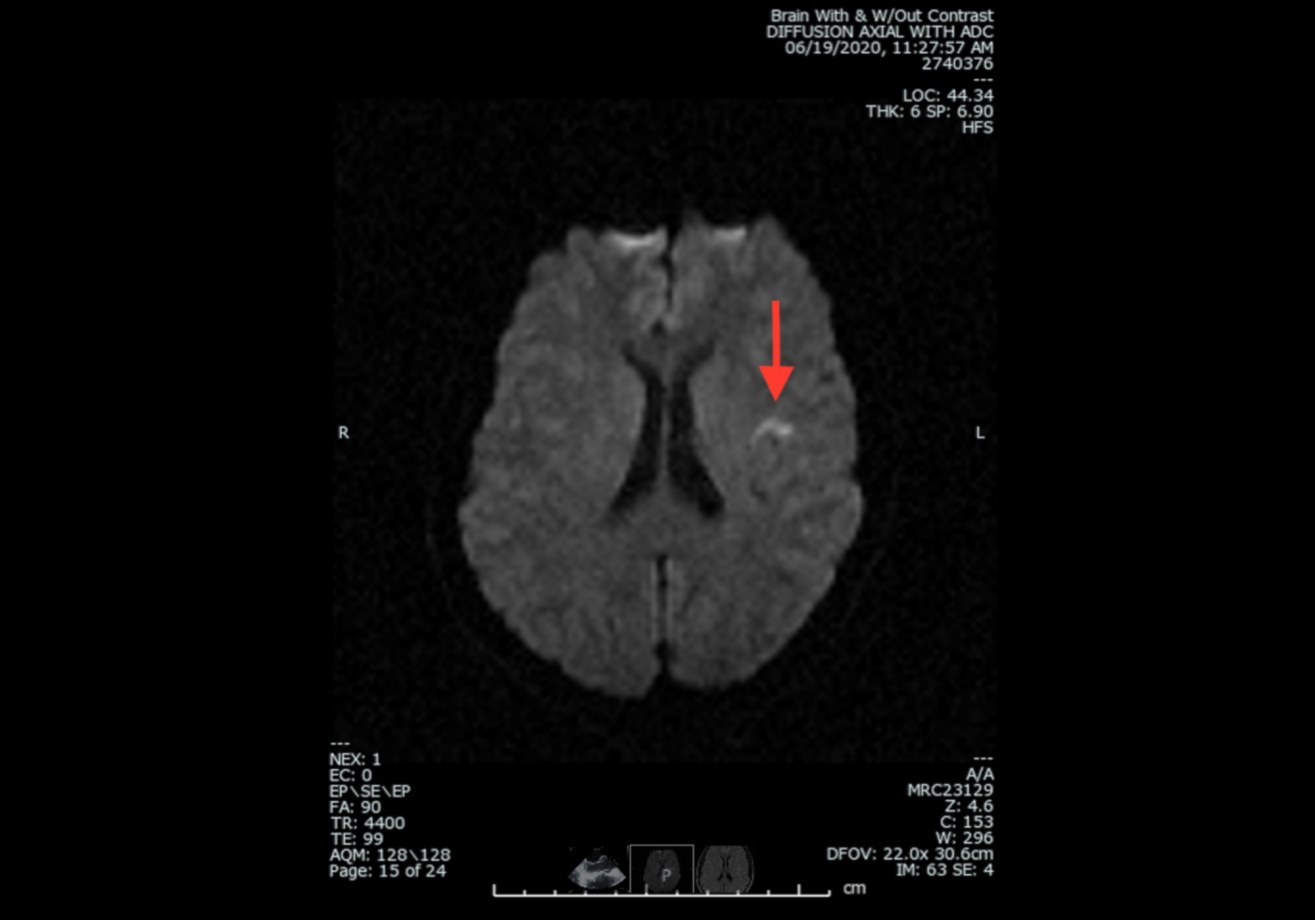
MRI head with and without contrast depicting a recent cortical infarct in left frontal region.

Lipid panel was significant for TG 133, HDL 47, LDL 78, TG 39, A1c of 5.0, and nonreactive rapid plasma reagin (RPR). Transthoracic echocardiogram (TTE) showed an ejection fraction (EF) of 55%-60% with left ventricular hypertrophy as well as increased thickness of the septal and posterior wall. As seen in Figure 2, MRI head with diffusion-weighted imaging showed a tiny curvilinear focus of restricted diffusion with subtle T2-FLAIR hyperintensity in the left frontal/insular region likely due to a recent cortical infarct without evidence for hemorrhage.

**Figure 3:**
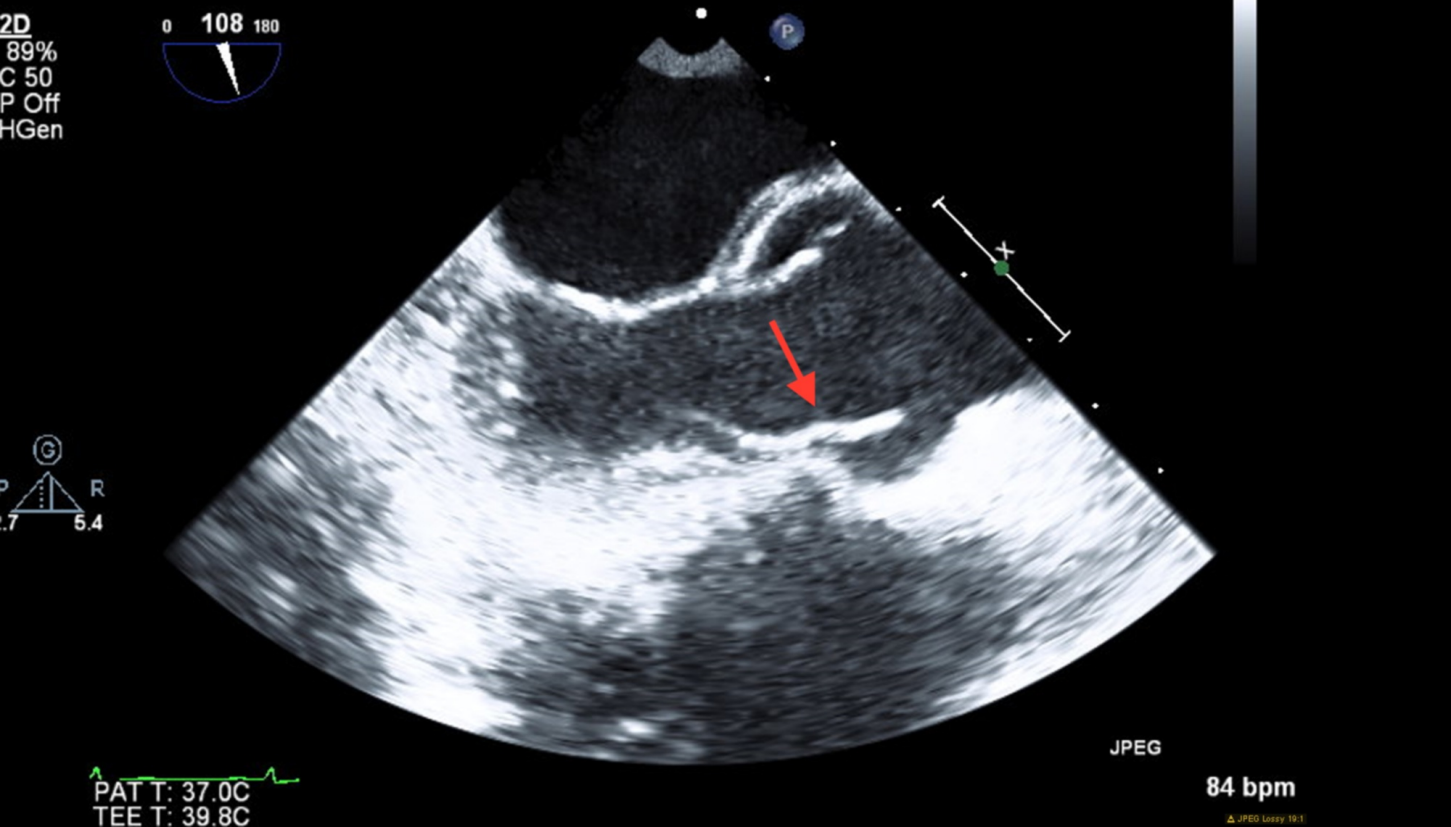
TEE depicting filiform lesion noted on aortic valve leaflet tips most likely LE. TEE, transesophageal echocardiogram; LE, Lambl's excrescence

Even though there was high suspicion that uncontrolled hypertension is the underlying cause of his stroke, a TEE was performed along with hypercoagulable workup to ensure no other etiologies were the cause of stroke given the patient's young age. TEE showed left ventricular EF to be 55%-60% with normal global left ventricular systolic function and filiform lesion noted on aortic valve leaflet tips most likely LE as seen in Figure 3. 

The patient was discharged on dual antiplatelet therapy with low-dose aspirin and clopidogrel for 21 days, high-intensity statin, antihypertensives and recommended to follow up with a Primary Care Provider and Cardiology within one week. 

## Discussion

Lambl's excrescences have been well reported as a possible rare cause of cryptogenic stroke as they can theoretically be a source of microthrombi after progressive wear and tear [3]. However, due to a paucity of cases, there have been no prospective studies that have established a causal relationship between LE and cardioembolic ischemic stroke. The only prospective study described in the literature is as per Roldan et al., who reported that there was no association between cardioembolic phenomenon and LE. In addition, they noted that the four-year rate of ischemic strokes was similar in those with and without LE [9]. However, this study had a small sample size, and due to low event rates, they were unable to exclude LE as a source of ischemic stroke definitively. 

Consequently, to state that LE is the source of cardioembolic phenomenon, it is prudent to rule out any other possible etiology [3]. Although our patient was noted to have elevated BP on admission, which is a known risk factor for ischemic events, LE's presence on the TEE and recent examination with normal BP further confounds the clinical picture. This is important as there are cases in the literature that report recurrent ischemic strokes in patients with LE [10]. In our patient, due to this theoretical increased risk of recurrent ischemic events, it was important not to completely exclude the clinical significance of LE regardless of other risk factors, especially given the young age. As a result, he was offered dual antiplatelet therapy in addition to aggressive risk factor modification, including strict BP control. The lack of clinical data on LE regarding its appropriate management is further detrimental to our patient's care, given the risk of increased recurrent ischemic stroke. Chu et al. recommend that patients with documented ischemic stroke and LE should have surveillance TEE at six months and one year after discharge to assess the stability of LE [3]. 

## Conclusions

We report a rare case of LE in a patient with ischemic stroke and underlying hypertension. This case illustrates and highlights the need for more prospective studies to establish a causal relationship between LE and cardioembolic strokes. This is essential as LE may cause recurrent ischemic events, which can possibly be detrimental leading to permanent disability. 
